# Immunoregulation on Mice of Low Immunity and Effects on Five Kinds of Human Cancer Cells of *Panax japonicus* Polysaccharide

**DOI:** 10.1155/2015/839697

**Published:** 2015-08-04

**Authors:** Zhang Jie, Li Chun-Yan, Li Jing-Ping, Guo Ren, Wang Hui, Pan Juan, Liu Sheng-Lan

**Affiliations:** ^1^Third Xiangya Hospital, Central South University, Changsha 410013, China; ^2^Changsha University, Changsha 410022, China; ^3^School of Pharmaceutical Science, Central South University, Changsha 410013, China; ^4^The Central Hospital of Taian, Taian 271000, China; ^5^Maternal and Child Health Hospital, Liuyang 410300, China

## Abstract

The goal of this study is to investigate the immunoregulative effects of *Panax japonicus* polysaccharide (PJPS) on mice of low immunity. An orthogonal experiment was designed to determine the best extraction process for PJPS. By the tests of macrophages swallow chicken red blood cells, Delayed-type hypersensitivity (DTH), and serum hemolysin value, we studied the immune adjustment ability of PJPS. MTT was employed to detect the effects of different concentrations of PJPS, respectively, in 24 h, 48 h, and 72 h on five kinds of human cancer cells. The results show that the best extraction process for PJPS was as follows: ratio of solvent consumption to raw material 40, extraction temperature 100°C, re-extracted two times, each extraction time 4 hours. PJPS can significantly improve the immune function of mice processed by cyclophosphamide and PJPS did not work on the above five cancer cells.

## 1. Introduction

*Rhizoma Panacis Japonici*, a special resource of Tujia nation in Hunan Province, belongs to Araliaceae plant and its dry rootstalk could be used as medicine. It is warm, sweet, and bitter. In Chinese medicine, being a natural plant used as both medicine and food, it has both the nutrition effects of ginseng and can activate blood circulation to dissipate blood stasis [1]. Several studies have indicated its pharmacological effects on the central nervous system [2, 3], digestive system [4], cardiovascular system [5, 6], immune system [7], inflammation [8–10], fatigue [11, 12], tumor [13, 14], and so on.

It contains various saponins, polysaccharides, and some active substances like amino acids, volatile oil [15], and so forth. Saponin is the most abundant material of chemical composition in the* Rhizoma Panacis Japonici*. At present, there are numerous studies concerning the chemical structure and pharmacological activity of saponins. However, the studies of* Panax japonicus* polysaccharide (PJPS) are limited. It may be partly due to the complex chemical structure and ambiguous mechanism of action. Ohtani et al. [16] reported that PJPS extracted from* Rhizoma Panacis Japonici* can activate the reticuloendothelial system, which suggests that PJPS can improve activity of macrophages in the reticuloendothelial system and play a role in immune regulation via this way. Other results suggest that the PJPS can promote immune organ weight index of immunosuppressed mice, significantly improve the spleen lymphocyte proliferation, promote serum IgM level following exposure to chicken red blood cells and QSH reaction, and promote natural killer's cells activity. It has better immune enhancement effect and recovers immune system of immunosuppressed mice [17].

## 2. Experimental

### 2.1. Extraction Process of PJPS

Firstly, single factor experiments were studied.* Rhizoma Panacis Japonici* powder, which has been sifted by 50 meshes, was extracted by hot water. The extraction rate of PJPS as index, the number of extractions, solvent ratio, extraction time, and extraction temperature were investigated as the single factors. Secondly, on the basis of single factor experiments, the orthogonal experiment was designed with the extraction rate of PJPS as index to optimize and determine the best extraction process. At last, validated experiment was made.

Selecting anhydrous glucose as the standard, this paper investigated the determination conditions and methodology of anthrone sulfuric acid method [18]. Consider

(1) the  extraction  rate  of  PJPS  (%)=the  quality  of  PJPS  extracted  from  Rhizoma  Panacis  Japonicithe  quality  of  PJPS  of  Rhizoma  Panacis  Japonici×100%.

### 2.2. Separations and Refining of PJPS

The aqueous extract, which was acquired according to the optimum extraction method of PJPS, was concentrated to a certain volume; then the concentrate was obtained. The concentrate was further precipitated by adding anhydrous ethanol to its concentration of 80% ethanol. It was refrigerated over 12 h. The sedimentation was separated by being centrifuged for 15 min (5000 rpm). Then, the sedimentation was washed twice with appropriate amount of ethanol and was freeze-dried into constant weight. Through these progresses, the insoluble substance was crude PJPS.

Crude PJPS solution, which was made up at the mass concentration of 1%, was added to 1.2% of papain solution (dynamic unit 400 *μ*/mg). Under the optimum pH value 6.0 and the optimum enzymatic hydrolysis temperature 40°C, enzymatic hydrolysis was reacted for 2 h. Enzyme deactivation was done at 90°C. This solution was centrifuged for 10 min (5000 rpm) to get rid of denatured protein, then the supernatant was obtained. The supernatant was precipitated for 12 h by the addition of ethanol to a final concentration of 80% (v/v), and centrifuged for 15 min (5000 rpm). The sedimentation was washed twice with appropriate amount of ethanol and was freeze-dried into constant weight. Through these progresses, the insoluble substance was refined PJPS.

The concentration of crude PJPS was 1%, and then 1.2% papain (dynamic unit 400 *μ*/mg) was added. In its optimum pH value of 6.0, the optimum enzymolysis temperature is 40°C, enzymatic hydrolysis was reacted for 2 h. Enzyme deactivation was done at 90°C. This solution was centrifuged for 10 min (5000 rpm) to get rid of denatured protein, then the supernatant was obtained. The supernatant was precipitated for 12 h by the addition of ethanol to a final concentration of 80% (v/v), and centrifuged for 15 min (5000 rpm). Sedimentation was washed twice with appropriate amount of ethanol and freeze-dried to constant weight. Through these progresses, the insoluble substance was refined PJPS.

### 2.3. The Immune Regulating Effect of PJPS in Mice with Immunosuppression

#### 2.3.1. Phagocytic Function of Peritoneal Macrophage [19]

According to the different treatment, 90 Kunming mice were randomly divided into normal control group, cyclophosphamide (CY) model control group, CY + lentinan group (positive control group), and CY + low, CY + middle, and CY + high dosage group by weight and gender. Every group has 15 mice. The drug groups were pretreated intragastrically with 100 mg/kg, 200 mg/kg, and 400 mg/kg PJPS. Positive control group was pretreated intragastrically with 150 mg/kg lentinan. Normal control group and cyclophosphamide model group were pretreated with saline at the same volume. All mice were separately administered once every day for 10 days for each group. From 7th day, cyclophosphamide was injected into enterocelia with 100 mg/kg for 3 days. At 11th day, mice were injected by 20% chicken red blood cell with 0.5 mL/only. Mice were executed after 35 minutes and cut open along the middle line of the abdominal wall skin, in which 2 mL physiological saline was injected by peritoneal injection. The experiment kneaded the mice's abdomen for 1 minute. Then abdominal lotion was sucked out, and then 1 drop of abdominal lotion was stilled on the glass slide and was observed by Wright-giemsa stain. Macrophages were counted 100 on each piece in the Oil immersion lens, and then the number of macrophages which swallowed chicken red blood cells and the number of chicken red blood cells which were swallowed were counted. The percentage of phagocytosis and phagocytic index was calculated. Consider
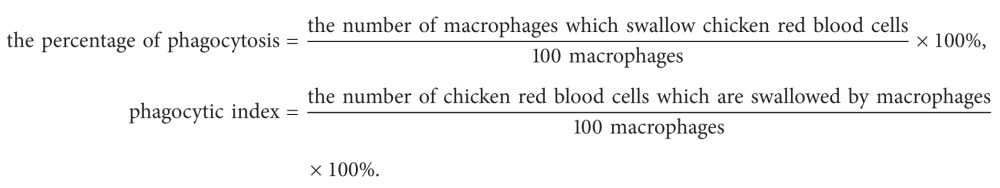
(2)At the same time in the count, the degree of chicken red blood cells to be digested determines macrophage phagocytosis and digestion ability and also determines the standards of phagocytosis, which is usually divided into four levels:

Class I: not be digested. Engulfed Chicken red blood cells are complete, cytoplasm is pale red or pale yellow with green, and cell nucleus is light purple.
Class II: mild digestion. Cytoplasm is chartreuse; cell nucleus is pycnosis and purplish blue.
Class III: serious digestion. Cytoplasm is dyed lightly and displays light grey.
Class  IV: completely digestion. A physalides similar to the size of chicken red blood cell can be seen in macrophages.

#### 2.3.2. Carbon Granular Clearance Ability

Group and administration were done like Section 2.3.1. On the 10th day after 2 h behind oral administration, each mouse was injected with India ink 0.1 mL/10 g that was diluted 5 times with saline via coccygeal vein. After 5 min and 15 min behind the injection, 60 *µ*L of blood plasma, respectively, was taken from the orbital venous plexus with Vacuum blood tube which was wet by heparin solution in advance in EP tube containing 6 mL 0.1% sodium carbonate solution. The EP tube was shaken well. Then their OD values were measured at 680 nm wavelength with the reagent blank tube zero. At last, mice were dislocated and executed and the immunologic effects were measured by clearance rate *K* of charcoal particles, phagocytic index *α*, and thymus and spleen index.

#### 2.3.3. Hemolysin Production Level

Group and administration were done like Section 2.3.1. From the fifth day, all groups were injected with 20% chicken red blood cell (CRBC) suspension of 0.2 mL. After the last treatment, the mice were killed for serum (2000 rpm, 10 min). Serum was diluted 500 times with saline water, to which 10% CRBC suspension of 0.5 mL and diluted guinea pig serum (complement) with 9 times saline water were added. By contrast, blank tube was added to saline 1 mL instead of mice serum liquid. Each tube in 37°C water bath is heated for 10 min and then is inserted in ice water bath for 10 min to terminate the reaction. After cooling, the tube was centrifuged at 2000 rpm for 10 min. Levels of serum hemolytic optical densities were measured in all groups. Results were analyzed by SPSS13.0 to compare differences between OD_540_ values of the groups.

#### 2.3.4. Delayed-Type Hypersensitivity (DTH) Induced by 2,4-Dinitrochlorobenzene (DNCB)

Group and administration were like Section 2.3.1. From 4th day, cyclophosphamide was injected into enterocelia with 100 mg/kg once.

*(1) Hypersensitive Response.* On 5th day, barium sulfide liquid was applied to the abdomen for unhairing. 50 *μ*L of 5% DNCB fluid by pipetting gun was spread on the surface of depilatory parts to cause hypersensitive response.

*(2) Checking Allergies.* On 10th day, 10 *μ*L of 1% DNCB solution was applied to mice in order to attack both sides of the left ear. At the same time, both sides of the right ear were coated with the same amount of acetone sesame oil as comparison. The mice were killed after 24 hours. The right and left ears were cut, which were punched 8 mm round hole. The wafers were weighed by a scale.

*(3) Results Analysis.* Comparing the weight of the different groups mice's left auricular and right auricular, experimenter analyzed the average weight difference by SPSS13.0.

#### 2.3.5. MTT Assay

Five kinds of cancer cells were chosen as the research object; they are human lung cancer cells HTB182, human colon cancer cells SW480, human kidney cancer cells HEK293, human nasopharyngeal carcinoma cell 5–8* F*, and human liver cancer cell HepG2. Briefly, the cancer cells were plated in 96-well plate at a density of 1000–10000 cells/well in their respective medium. The cells were then incubated at 37°C in a 5% CO_2_ environment for 24 h. The experiment was divided into normal control group, negative control group, positive control group (DDP), and PJPS different dosage groups. Every group had 6 wells. After 24 hours, the normal control group and the negative control group were joined with the culture medium without drugs and the positive control group was joined with the culture medium containing 5~10 *μ*g/mL DDP (cis-Dichlorodiammineplatinum(II); each cell had its cisplatin sensitivity and actual operation of each cell should choose a suitable concentration). The final concentration of PJPS different dosage groups was, respectively, 50 *μ*g/mL, 100 *μ*g/mL, 200 *μ*g/mL, 400 *μ*g/mL, and 800 *μ*g/mL and was, respectively, incubated at 37°C in a 5% CO_2_ environment for 24 h, 48 h, and 72 h. After the designated time period, 20 *μ*L of 3-(4.5-dimethythiazol-2-yl)-2,5-diphenyl tetrazolium bromide was added to each well and the plates were incubated at 37°C for additional 4 h. The formazan crystals formed in the wells were dissolved in 150 *μ*L DMSO. The absorbance was measured at 490 nm using ELISA. The OD490 values were analyzed with SPSS13.0 statistical analysis and were compared with the difference.

## 3. Results

On the basis of the results of single factor experiments, designed L_9_ (3^4^) orthogonal test of using the extraction rate of PJPS as index and using amount of solvent, extraction time, and extraction times as factors aimed to determine the rational extraction process. Analysis table of orthogonal experiment results is shown in Table 1 and variance analysis is shown in Table 2.

The range in Table 1 shows that the order of the influence factors on extraction rate of PJPS is *C* (extraction temperature) >*A* (solvent ratio) >*B* (time). The results of analysis of variance in Table 2 showed that the solvent ratio and extraction temperature have a statistic significant influence on extraction rate of PJPS while extraction time on the influence of extraction rate of PJPS has no statistical significance. Through *K* values of other factors and orthogonal experiment result, we can find that the best extraction technology of PJPS is *A*_3_ *B*_2_ *C*_3_. The best extraction process for PJPS is demonstrated as follows: the solvent consumption was 40 times, the steeping time was 40 min, and the extraction temperature was 100°C, re-extracted at two times and each extraction time is 4 hours.

### 3.1. Validation Experiments

Table 3 shows that extraction rates of PJPS were 97.99%, 98.35%, and 97.73%, RSD was 0.38%, and the average extraction rate reached 98.36%. It shows that the extraction technology is stable and reliable in gaining high extraction rate.

### 3.2. Macrophage Cell Swallowed the Chicken Red Blood Cells

**Figure 1:**
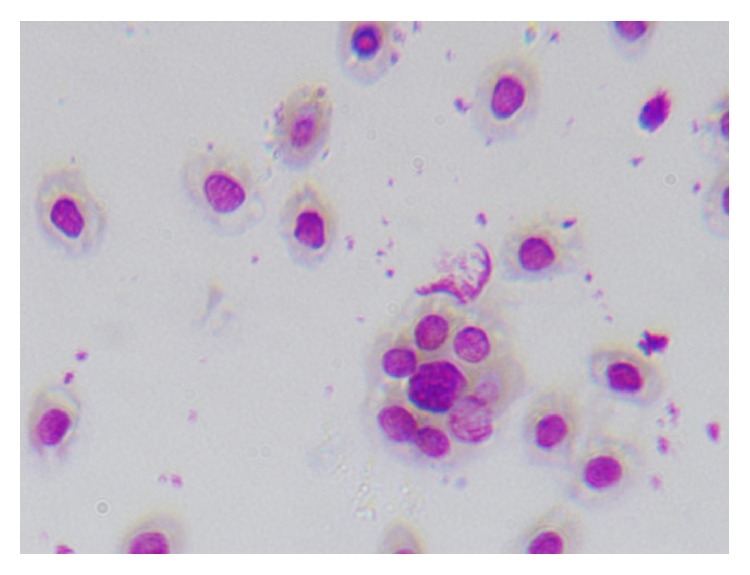
Macrophages adsorbed multiple chicken red blood cells.

**Figure 2:**
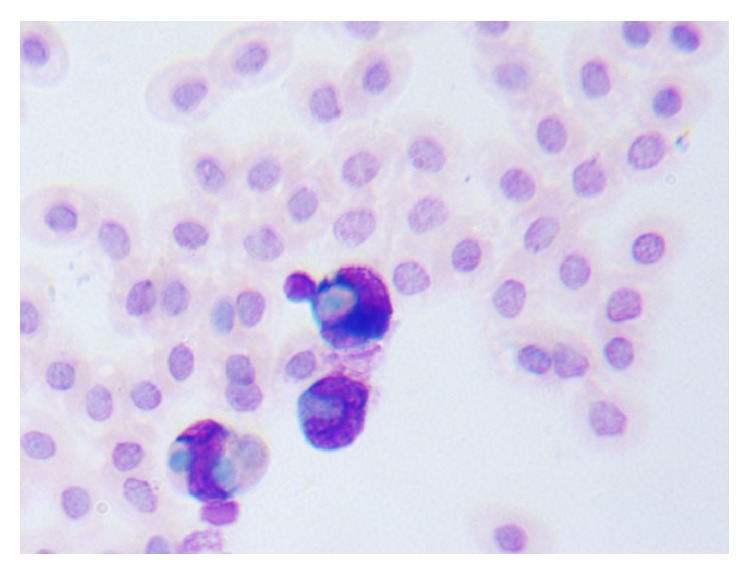
Macrophages swallowed multiple chicken red blood cells.

**Figure 3:**
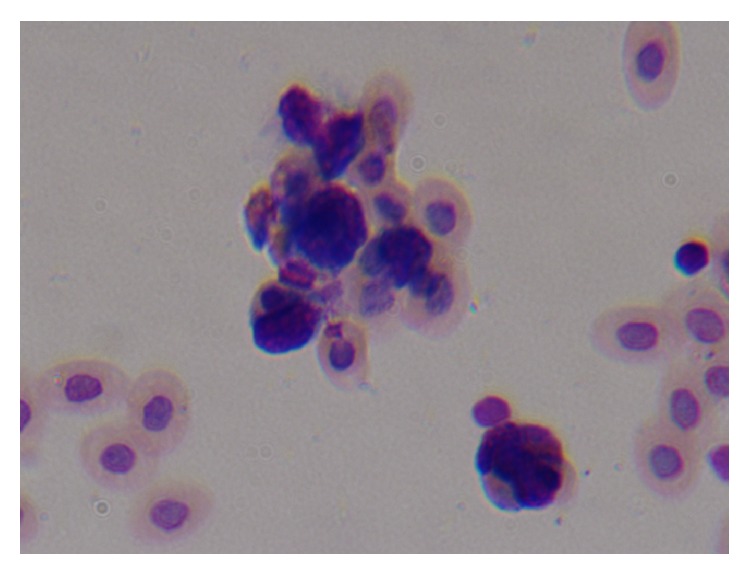
Macrophages adsorbed and swallowed multiple chicken red blood cells.

The morphological changes of macrophages during the process, in which microphages swallowed chicken erythrocytes, were observed by oil immersion lens. Figures 1, 2, and 3 are several typical cases. The macrophage phagocytic functions in the experiment of peritoneal macrophage phagocytizing chicken red blood cell are showed in Table 4.

In comparison with the control group, phagocytic rate and phagocytic index in CY group were significantly reduced. The results showed that CY could effectively decrease the mouse's peritoneal macrophage phagocytosis. Compared with CY group, phagocytic rate and phagocytic index in positive drug group were significantly enhanced (*P* < 0.01). The phagocytic rate and phagocytic index in middle (200 mg·kg^−1^) and high dose (400 mg·kg^−1^) of PJPS groups were promoted greatly compared to the CY group (*P* < 0.05), which indicated that the middle and high dose of PJPS could recover phagocytosis function of immunosuppression mice caused by CY. Lentinan group had no significant differences compared to the high dose group of PJPS.

### 3.3. Carbon Clearance Test

In comparison with the normal group, carbon clearance index *K* and Phagocytic index *α* in CY group were significantly reduced. The results showed that CY could effectively decrease the mouse's peritoneal macrophage phagocytosis. In addition, high dose (400 mg·kg^−1^) of PJPS group had no significance compared with normal group, which indicated that PJPS could make the mouse's phagocytosis function return back to normal. Compared with CY group, carbon clearance index *K* in positive drug group and high dose (400 mg·kg^−1^) of PJPS groups were significantly enhanced (*P* < 0.05). Lentinan group (*P* < 0.01) and middle (200 mg·kg^−1^) (*P* < 0.05) and high dose (400 mg·kg^−1^) PJPS groups (*P* < 0.01) were promoted greatly compared to the CY group, which indicated that lentinan group and the middle and high dose of PJPS could recover the phagocytosis function of immunosuppression mice caused by CY. The influences of PJPS on the carbon clearance index *K* ^1/2^ and Phagocytic index *α* were showed in the Table 5.

### 3.4. Delayed-Type Hypersensitivity (DTH) Induced by DNCB

In comparison with the normal group, auricle swelling degree in CY group and all dosage groups of PJPS were significantly enhanced. Compared with CY group, lentinan group has statistical significance (*P* < 0.01). In comparison with the normal group, the spleen index and thymus index were decreased significantly in CY group (*P* < 0.01). The influences of PJPS on the auricle swelling degree and visceral index were showed in the Table 6. It showed that the immune suppression model that were induced via intraperitoneal injection of CY was established successfully. Compared with CY group, the spleen index in lentinan group and PJPS high dosage group were increased significantly (*P* < 0.05). The thymus index in both lentinan group (*P* < 0.01) and high dose group of PJPS (*P* < 0.05) was increased significantly compared to the CY group. Lentinan group had no significant differences compared to the high dose group of PJPS.

### 3.5. Hemolysin Production Level

By comparing the difference of each OD_540_ values in Table 7, it can be found that OD_540_ values of groups were significantly lower than normal control group. CY group, Lentinan group, low dose group, and middle dose group compared with normal control group also had extremely significant difference (*P* < 0.01). Compared with CY group, OD_540_ values of PJPS different dosage groups were significantly higher than the CY group (*P* < 0.01), and with the increase of the dose of PJPS, OD_540_ values had a tendency to increase but had no statistical difference between PJPS different dosage groups. OD_540_ value of the high dose group was obviously higher than that of lentinan group (*P* < 0.05).

### 3.6. The Influence of PJPS on Proliferation of the Five Kinds of Human Cancer Cells in Tables 8, 9, 10, 11, and 12

The results showed that the OD value of DDP group in 24 h, 48 h, and 72 h was lower than that of negative control group (*P* < 0.05), but all different doses of PJPS groups have no significant differences compared with negative control group. The result showed that PJPS had no obvious proliferation inhibition for five kinds of cancer cells.

## 4. Discussions

The orthogonal test was used to optimize the extraction conditions of polysaccharides from the rhizomes of* Panax japonicus* and elevate the extraction rate of PJPS. In the result, the best extraction process of PJPS is demonstrated as follows: the solvent consumption was 40 times, the steeping time was 40 min, the extraction temperature was 100°C, re-extraction at two times and each extraction time is 4 hours. All of PJPS were heteropolysaccharide, and polysaccharides with different ethanol concentration consist of different components. Polysaccharides contain pyranose ring, and the sugar composition analysis showed that PJPS are composed of arabinose, glucose, and galactose and the content of arabinose increased with the increasing of ethanol concentration [20, 21]. In activity analysis, polysaccharides with different ethanol concentration display different levels in activity, and polysaccharides with higher ethanol concentration have stronger recovery effect on DNA impaired [22].

As is well known, chemotherapy treatments for malignant tumour have strong adverse reactions; meanwhile myelosuppression and immunosuppression are especially severe and common. These toxic and side effects cause numerous patients to get infected and die of serious infectious diseases [23]. Polysaccharides have activity in regulation of body's immunity, which draw the attentions of domestic and international scholars, and some polysaccharides or extracts mainly containing polysaccharides have been further taken to clinical assessment in humans, such as polysaccharides from mushrooms. At present, the anticancer properties of polysaccharides have been shown to be primarily mediated via three approaches of direct cytotoxicity, immunoenhancement, and synergistic effects in combination with anticancer drugs. Meanwhile, the synergistic effects are known to be mediated by enhancing the sensitivity of tumour and elevating immune response to treatments [24]. Our experiments indicate that PJPS have effects on resisting immunosuppression caused by CY, such as improving macrophage phagocytosis and boosting the level of IgM in plasma. Some researches indicated that PJPS have the haematopoietic effect, repair impaired DNA, and have antioxidant activities [20, 22, 23, 25, 26]. Even though MTT assay indicated that PJPS do not have directly obvious effects on proliferation of lung cancer cells HTB182, colon cancer cells SW480, kidney cancer cells HEK293, nasopharyngeal carcinoma cella (NPC) 5–8* F*, and human liver cancer cells HepG2, we can utilize haematopoietic effect and immunoenhancement of PJPS to resist myelosuppression and immunosuppression of chemotherapy; in other words, we can use PJPS in combination with anticancer drugs in clinic. Through this way, the risk of infection and myelosuppression may be decreased, as well as adverse effects, psychological and economical decline of using antibiotics; consequently PJPS in combination with chemotherapy may reduce mortality of cancer patients. However, this hypothesis needs to be proved by more studies. The mechanisms of PJPS resisting myelosuppression and immunosuppression are also supposed to be further confirmed.

## 5. Conclusion

PJPS can significantly improve the immune function of mice processed by cyclophosphamide.

## Figures and Tables

**Table 1:** Results of orthogonal test for extraction technique of PJPS (%).

Number	*A*	*B*	*C*	*D*	Extraction rate of PJPS (%)
1	1	1	1	1	65.66
2	1	2	2	2	73.68
3	1	3	3	3	86.55
4	2	1	2	3	75.36
5	2	2	3	1	93.21
6	2	3	1	2	68.23
7	3	1	3	2	97.82
8	3	2	1	3	79.97
9	3	3	2	1	77.44
*K*1	75.297	79.613	71.287	78.757	
*K*2	78.933	82.287	75.480	79.910	
*K*3	85.063	77.393	92.527	80.627	
*R*	9.766	4.894	21.240	1.870	

**Table 2:** Analysis of variance.

Factors	Sum of squares	DOF	*F*	*F* critical value	Significance
*A*	146.190	2	27.371	19.000	∗
*B*	36.020	2	6.744	19.000	
*C*	759.310	2	142.166	19.000	∗
Error	5.341	2			

*Note*.  ^∗^ *P* < 0.05.

**Table 3:** Results of validation experiments.

Sample	1	2	3	Average	RSD (%)
Extraction rate (%)	97.99	98.35	98.73	98.36	0.38

**Table 4:** The influence of PJPS on peritoneal macrophage phagocytizing chicken red blood cell.

Groups	Phagocytic rate (%)	Phagocytic index (%)
Normal group	16.36 ± 0.81	19.64 ± 0.93
CY group	9.00 ± 0.49 ^∗∗^	10.31 ± 0.44 ^∗∗^
Lentinan group	14.31 ± 0.66 ^∗##^	16.92 ± 0.64 ^∗∗##^
PJPS low dosage group	9.07 ± 0.50 ^∗∗△△^	10.54 ± 0.49 ^∗∗△△^
PJPS middle dosage group	10.85 ± 0.63 ^∗∗#△△^	12.38 ± 0.79 ^∗∗#△△^
PJPS high dosage group	13.76 ± 0.74 ^∗∗##^	15.92 ± 0.74 ^∗∗##^

Note: values represent mean ± SEM; *n* = 15; significance as per Student's *t*-test compared with normal control group;  ^∗^ *P* < 0.05;  ^∗∗^ *P* < 0.01, compared with CY group;  ^#^ *P* < 0.05;  ^##^ *P* < 0.01, compared with lentinan group;  ^△△^ *P* < 0.01.

**Table 5:** The influence of PJPS on the carbon clearance index *K* and Phagocytic index *α*.

Groups	Carbon clearance index *K* ^1/2^	Phagocytic index *α*
Normal group	0.146 ± 0.026	4.170 ± 0.464
CY group	0.059 ± 0.013 ^∗∗^	2.071 ± 0.334 ^∗∗^
Lentinan group	0.099 ± 0.011 ^∗#^	3.264 ± 0.219 ^∗##^
PJPS low dosage group	0.084 ± 0.013 ^∗∗^	2.817 ± 0.306 ^∗∗^
PJPS middle dosage group	0.096 ± 0.006 ^∗^	3.111 ± 0.145 ^∗#^
PJPS high dosage group	0.101 ± 0.008 ^∗#^	3.379 ± 0.221 ^##^

Note: values represent mean ± SEM; *n* = 15; significance as per Student's *t*-test compared with normal control group;  ^∗^ *P* < 0.05;  ^∗∗^ *P* < 0.01, compared with CY group;  ^#^ *P* < 0.05;  ^##^ *P* < 0.01.

**Table 6:** The influence of PJPS on the auricle swelling degree and visceral index.

Groups	Auricle swelling degree (mg)	Thymus index (mg/g)	Spleen index (mg/g)
Normal group	3.08 ± 0.47	3.20 ± 0.21	6.04 ± 0.44
CY group	5.31 ± 0.66 ^∗∗^	1.77 ± 0.13 ^∗∗^	3.67 ± 0.35 ^∗∗^
Lentinan group	3.08 ± 0.42 ^##^	2.54 ± 0.11 ^∗∗##^	5.15 ± 0.34 ^∗##^
PJPS low dosage group	5.07 ± 0.47 ^∗△^	2.06 ± 0.23 ^∗∗△^	3.86 ± 0.38 ^∗∗△^
PJPS middle dosage group	4.62 ± 0.46 ^∗△^	2.17 ± 0.18 ^∗∗^	4.09 ± 0.36 ^∗∗^
PJPS high dosage group	4.46 ± 0.66 ^∗△^	2.28 ± 0.15 ^∗∗#^	4.72 ± 0.31 ^∗∗#^

Note: values represent mean ± SEM; *n* = 15; significance as per Student's *t*-test compared with normal control group;  ^∗^ *P* < 0.05;  ^∗∗^ *P* < 0.01, compared with CY group;  ^#^ *P* < 0.05;  ^##^ *P* < 0.01, compared with lentinan group;  ^△^ *P* < 0.05.

**Table 7:** The influence of PJPS on the OD_540 _value.

Groups	OD_540_ value
Normal group	0.108 ± 0.001
CY group	0.090 ± 0.001 ^∗∗^
Lentinan group	0.099 ± 0.003 ^∗∗##^
PJPS low dosage group	0.098 ± 0.003 ^∗∗##^
PJPS middle dosage group	0.098 ± 0.001 ^∗∗##^
PJPS high dosage group	0.103 ± 0.001 ^∗##△^

Note: values represent mean ± SEM; *n* = 15; significance as per Student's *t*-test compared with normal control group;  ^∗^ *P* < 0.05;  ^∗∗^ *P* < 0.01, compared with CY group;  ^##^ *P* < 0.01, compared with lentinan group;  ^△^ *P* < 0.05.

**Table 8:** The influence of PJPS on human nasopharyngeal carcinoma cell 5-8F proliferation in different time.

Groups	Drug concentration	24 h	48 h	72 h
	(*μ*g/mL)	OD_490_ value	OD_490_ value	OD_490_ value
Negative group	—	0.494 ± 0.019	0.598 ± 0.016	0.675 ± 0.017

Different dosage groups of PJPS	50	0.478 ± 0.017	0.580 ± 0.020	0.694 ± 0.013
	100	0.492 ± 0.018	0.575 ± 0.018	0.676 ± 0.013
	200	0.504 ± 0.024	0.584 ± 0.012	0.680 ± 0.018
	400	0.508 ± 0.025	0.590 ± 0.021	0.683 ± 0.010
	800	0.484 ± 0.035	0.597 ± 0.019	0.687 ± 0.018

DDP group	5	0.401 ± 0.016 ^∗∗^	0.404 ± 0.023 ^∗∗^	0.325 ± 0.027 ^∗∗^

Note: values represent mean ± SEM; *n* = 6; significance as per Student's *t*-test compared with negative group;  ^∗∗^ *P* < 0.01.

**Table 9:** The influence of PJPS on lung cancer cells HTB182 proliferation in different time.

Groups	Drug concentration	24 h	48 h	72 h
	(*μ*g/mL)	OD_490_ values	OD_490_ values	OD_490_ values
Negative group	—	0.474 ± 0.015	0.536 ± 0.015	0.590 ± 0.014

Different dosage groups of PJPS	50	0.496 ± 0.018	0.522 ± 0.018	0.594 ± 0.015
	100	0.482 ± 0.016	0.513 ± 0.013	0.589 ± 0.015
	200	0.500 ± 0.015	0.510 ± 0.015	0.611 ± 0.021
	400	0.484 ± 0.011	0.516 ± 0.019	0.601 ± 0.014
	800	0.495 ± 0.011	0.514 ± 0.018	0.584 ± 0.024

DDP group	10	0.376 ± 0.015 ^∗∗^	0.405 ± 0.016 ^∗∗^	0.396 ± 0.018 ^∗∗^

Note: values represent mean ± SEM; *n* = 6; significance as per Student's *t*-test compared with negative group;  ^∗∗^ *P* < 0.01.

**Table 10:** The influence of PJPS on kidney cancer cells HEK293 proliferation in different time.

Groups	Drug concentration	24 h	48 h	72 h
	(*μ*g/mL)	OD_490_ values	OD_490_ values	OD_490_ values
Negative group	—	0.376 ± 0.021	0.543 ± 0.018	0.662 ± 0.012

Different dosage groups of PJPS	50	0.402 ± 0.015	0.581 ± 0.009	0.666 ± 0.011
	100	0.403 ± 0.011	0.592 ± 0.012	0.662 ± 0.015
	200	0.375 ± 0.016	0.594 ± 0.017	0.669 ± 0.014
	400	0.399 ± 0.013	0.595 ± 0.014	0.664 ± 0.007
	800	0.377 ± 0.011	0.588 ± 0.013	0.662 ± 0.011

DDP group	10	0.266 ± 0.016 ^∗∗^	0.363 ± 0.019 ^∗∗^	0.438 ± 0.007 ^∗∗^

Note: values represent mean ± SEM; *n* = 6; significance as per Student's *t*-test compared with negative group;  ^∗∗^ *P* < 0.01.

**Table 11:** The influence of PJPS on colon cancer cells SW480 proliferation in different time.

Groups	Drug concentration	24 h	48 h	72 h
	(*μ*g/mL)	OD_490_ values	OD_490_ values	OD_490_ values
Negative group	—	0.494 ± 0.030	0.490 ± 0.019	0.601 ± 0.013

Different dosage groups of PJPS	50	0.440 ± 0.020	0.491 ± 0.011	0.600 ± 0.011
	100	0.418 ± 0.017	0.500 ± 0.01	0.599 ± 0.009
	200	0.431 ± 0.023	0.496 ± 0.01	0.595 ± 0.011
	400	0.436 ± 0.033	0.497 ± 0.014	0.599 ± 0.013
	800	0.428 ± 0.037	0.495 ± 0.011	0.610 ± 0.012

DDP Value	20	0.307 ± 0.023 ^∗∗^	0.403 ± 0.018 ^∗∗^	0.467 ± 0.017 ^∗∗^

Note: values represent mean ± SEM; *n* = 6; significance as per Student's *t*-test compared with negative group;  ^∗∗^ *P* < 0.01.

**Table 12:** The influence of PJPS on human liver cancer cells HepG2 proliferation in different time.

Groups	Drug concentration	24 h	48 h	72 h
	(*μ*g/mL)	OD_490_ values	OD_490_ values	OD_490_ values
Negative group	—	0.440 ± 0.023	0.588 ± 0.017	0.689 ± 0.022

Different dosage groups of PJPS	50	0.473 ± 0.019	0.608 ± 0.019	0.678 ± 0.022
	100	0.471 ± 0.017	0.601 ± 0.019	0.702 ± 0.023
	200	0.461 ± 0.028	0.613 ± 0.020	0.689 ± 0.026
	400	0.488 ± 0.021	0.605 ± 0.013	0.685 ± 0.017
	800	0.385 ± 0.015	0.564 ± 0.015	0.698 ± 0.023

DDP Group	10	0.354 ± 0.018 ^∗∗^	0.351 ± 0.019 ^∗∗^	0.347 ± 0.017 ^∗∗^

Note: values represent mean ± SEM; *n* = 6; significance as per Student's *t*-test compared with negative group;  ^∗∗^ *P* < 0.01.
